# A systematic literature review on digital literacy, employability, and innovative work behavior: emphasizing the contextual approaches in HRM research

**DOI:** 10.3389/fpsyg.2024.1448555

**Published:** 2025-01-17

**Authors:** Angela Caroline, Martine J. H. Coun, Agus Gunawan, Jol Stoffers

**Affiliations:** ^1^Faculty of Management, Open University of the Netherlands, Heerlen, Netherlands; ^2^Department of Business Administration, Faculty of Social and Political Sciences, Parahyangan Catholic University, Bandung, Indonesia; ^3^Research Centre for Employability, Zuyd University of Applied Sciences, Sittard, Netherlands; ^4^Research Centre for Education and the Labour Market (ROA), Maastricht University, Maastricht, Netherlands

**Keywords:** digital literacy, employability, innovative work behavior, systematic literature review, bibliometric analysis, contextual approaches

## Abstract

Knowledge Society 5.0 and Industry 5.0 require workers with adaptable employability skills and who engage in innovative work behavior that help companies to create innovative products and processes that are difficult for competitors to imitate. Extant research examines employability, and innovative work behavior, but there are still few articles that include digital literacy in their study. In fact, digital literacy is closely related to human resources in the new workforce whose daily activities are closely related to digital technology. Through bibliometric analysis and a systematic literature review of the interplay among digital literacy, employability, and innovative work behavior we synthesize research trends, measurements, theoretical frameworks, and conceptual models on these topics. In addition, some contextual considerations will be utilized to ensure accurate data interpretation. Findings suggest that there is no generic measure of digital literacy, especially in business contexts, that links this concept to either employability or innovative work behavior. Digital literacy is particularly important to increase employability and stimulate both innovative behavior and performance. Future research should explore these topics using various methodologies and theoretical frameworks, combining them with multiple perceptions across workers and countries, especially considering the pace of technological development.

## 1 Introduction

The digital age encompasses both Knowledge Society 5.0 and Industry 5.0, a transformative period characterized by rapid technological progress and ubiquitous digital technologies (Henderikx and Stoffers, 2022; Phillips et al., 2017) which presents opportunities and challenges to both organizations and individuals (Hngoi et al., 2023). Industry 5.0, a human-centric extension of Industry 4.0 (Nousala and Metcalf, 2024), requires a workforce skilled in advanced digital technologies and a deep understanding of emerging technologies (Maddikunta et al., 2022). Fueled by AI, robotics, IoT, and big data, this paradigm shift is reshaping the business world (Adel, 2022). Knowledge Society 5.0, on the other hand, emphasizes the creation, sharing, and application of knowledge and innovation as key drivers of economic growth and societal development (Troisi et al., 2023).

To remain competitive and relevant in this volatile, uncertain, complex, and ambiguous (VUCA) landscape, organizations and individuals must deal with complex interplays among knowledge, digital literacy (DL), and technological innovation. By doing so, they can harness the power of knowledge and technology to stay competitive in the evolving market.

The digital era triggered modifications to organizational models and heightened workplace diversity by reshaping task execution (Phillips et al., 2017). This era also led to a profound evolution in the workplace, replacing traditional job roles and creating new ones (Van Laar et al., 2017). This shift has presented a new challenge for current and future workers: finding and maintaining jobs in a labor market that increasingly prioritizes individuals with higher knowledge or higher-order thinking skills. This shift in the labor market has profound implications for employability. Employability is employees’ ability to acquire and maintain a job (Habets et al., 2021) by continuously increasing knowledge, skills, and attitudes (Van der Heijden et al., 2018) so they can adapt to changing times and labor market demands (Stoffers et al., 2020a; Hoedemakers et al., 2023). Individuals who possess these skills are more likely to secure and maintain stable employment in an increasingly competitive job market. In today’s digital workplace, workers are pushed to have digital literacies to accommodate the digital technologies. DL underlines the need for proficiency in using digital technology devices when accessing and searching for information, and with regard to the protection of personal data, privacy and problem-solving (Schauffel et al., 2021; Ferrari, 2013). DL is more than skills in a digital environment (Van Laar et al., 2017); it can enable employees to communicate and collaborate effectively, build professional networks, and efficiently access information such as searching for job opportunities, upskilling based on online resources to meet changing workforce need. Previous research also show that DL enhances employability (Tinmaz et al., 2022; Schauffel et al., 2021). This ability gives a higher chance for both future but also present workers to have a good work opportunity in this digital era.Organization’s survival depends on their ability to deliver cutting-edge solutions consistently (Henderikx and Stoffers, 2022), and therefore they must not only adapt but also innovate products proactively (Stoffers et al., 2020a). To make an innovative product, workers are now required to have updated skillsets that align with industry requirements (Van der Heijden et al., 2018) and result in innovative performance that ensures distinctiveness and continuous organizational development (Stoffers et al., 2020b). This condition relates to innovative work behavior (IWB) that denotes an individual’s capacity to initiate, promote, and execute fresh ideas, goods, and services (Janssen, 2000; De Jong and Den Hartog, 2010), thereby fostering the organization’s innovative abilities (Stoffers et al., 2020c). When previous research emphasizes the need for competence-based antecedents of IWB, we acknowledge that DL is one of the important skills for supporting IWB. DL enables individuals to acquire new knowledge from digital media, creatively solve problems through critical evaluation of online information and collaborative insights and contribute to innovative products. DL can help employees for keeping up with developments and innovating product, process and services (Van Laar et al., 2017).

Complex interplays among DL, employability, and IWB are paramount in contemporary digital workplaces. As the new workforce is deeply intertwined with digital technology during daily activities, DL not only enhances employees’ skills and IWBs but also contributes to organizational innovation (Phillips et al., 2017). Both employability and IWB are part of the HR (Human Resource) outcome. As HR outcomes are closely linked to their specific context (Farndale et al., 2023), the concept of contextual approach has gained unprecedented significance (Hoedemakers et al., 2023). Context can be seen as a set of factors that directly or indirectly surround and affect the phenomenon. Context not only influences the relationship between variables in HR topics but also serves as a lens through which we can extract meaning and discern the underlying reasons for relational dynamics (Farndale et al., 2023). Some researchers also highlighted the importance of contextual research in digital literacy such as different technologies, industries, and/or groups (Tinmaz et al., 2022).

Previous research shows that there is a need for a correlation of DL with HR variables (Audrin et al., 2024). In this regard, employability and innovative work behavior are two pivotal factors in the contemporary workplace (Hapsari et al., 2019). To address the limitations of previous research, this study used a bibliometric analysis to reveal more information on how DL, DL and employability, and DL and IWBs are related or centered on what major topics (Donthu et al., 2021; Tinmaz et al., 2022). We use a systematic approach to identify, analyze, and synthesize findings from existing research (Donthu et al., 2021), limited to empirical studies that assess relationships among DL, employability, and IWBs. We explore methodologies, measurements, variables in conceptual models, theoretical frameworks, and research findings to gain a comprehensive understanding of the topic.

The contribution of this study is twofold. First, findings on these topics contribute to a comprehensive understanding of the interplay among DL, employability, and IWBs, particularly in management and business research. Second, this study offers a research agenda and conceptual model that can be used in future research. This study also has practical implications to organizations, policymakers, and educators on bridging skill gaps and fostering a sustainable, innovative workforce in the digital era.

## 2 Preliminary study: a bibliometric analysis

### 2.1 Methods

Bibliometric analysis was conducted to discover trends and interrelationships among topics to identify research gaps and generate new ideas for future research (Donthu et al., 2021). Table 1 shows five inclusion criteria for bibliometric analysis. Synonyms were necessary to expand the search. We focused on articles published in English to get a deeper understanding of the content of the articles. We incorporated only articles that were published until December 31, 2022. Peer-reviewed journal articles from the Web of Science (WoS) database were selected due to their detailed classifications (i.e., categories), published status, and high-impact journal collection (Caputo et al., 2021). These criteria were used to ensure data consistency and reliability of results and to facilitate the researchers’ rechecking of data.

**Table 1:** Inclusion and exclusion criteria during publication searches in databases for bibliometric analysis.

	Inclusion	Exclusion
Terms/keywords	Digital literacy: “digital literacy” OR “digital skills” OR “digital competence” OR “digital fluency” Employability: “employability” OR “perceive employability” Innovative work behavior: “innovation” OR “innovative (work) behavior*” OR “idea generation” OR “idea promotion” OR “idea implementation” OR “idea creation” OR “idea realization”	Other terms/keywords
Language	English	Non-English
Time	From unknown to December 2022	After December 2022
Document type	Journal articles	Conference papers, books, book chapters, and news articles
Databases	WoS	Elsevier, Emerald, etc.

Bibliometric analysis falls into two categories: objective (performance analysis) and subjective (science mapping) (Donthu et al., 2021). We used two performance analyses—publication-related metrics and citation-related metrics provided by WoS to assess the extent of current scientific research on DL, DL and employability, and DL and IWB.

We used co-word analysis to discover relationships among topics and identify thematic clusters and foundational topics (Caputo et al., 2021). We used the VOS viewer to construct and visualize bibliometric networks (Van Eck and Waltman, 2018) between terms (i.e., DL, DL-employability, and DL-IWB).

### 2.2 Results

#### 2.2.1 Performance analysis

**Figure 1:**
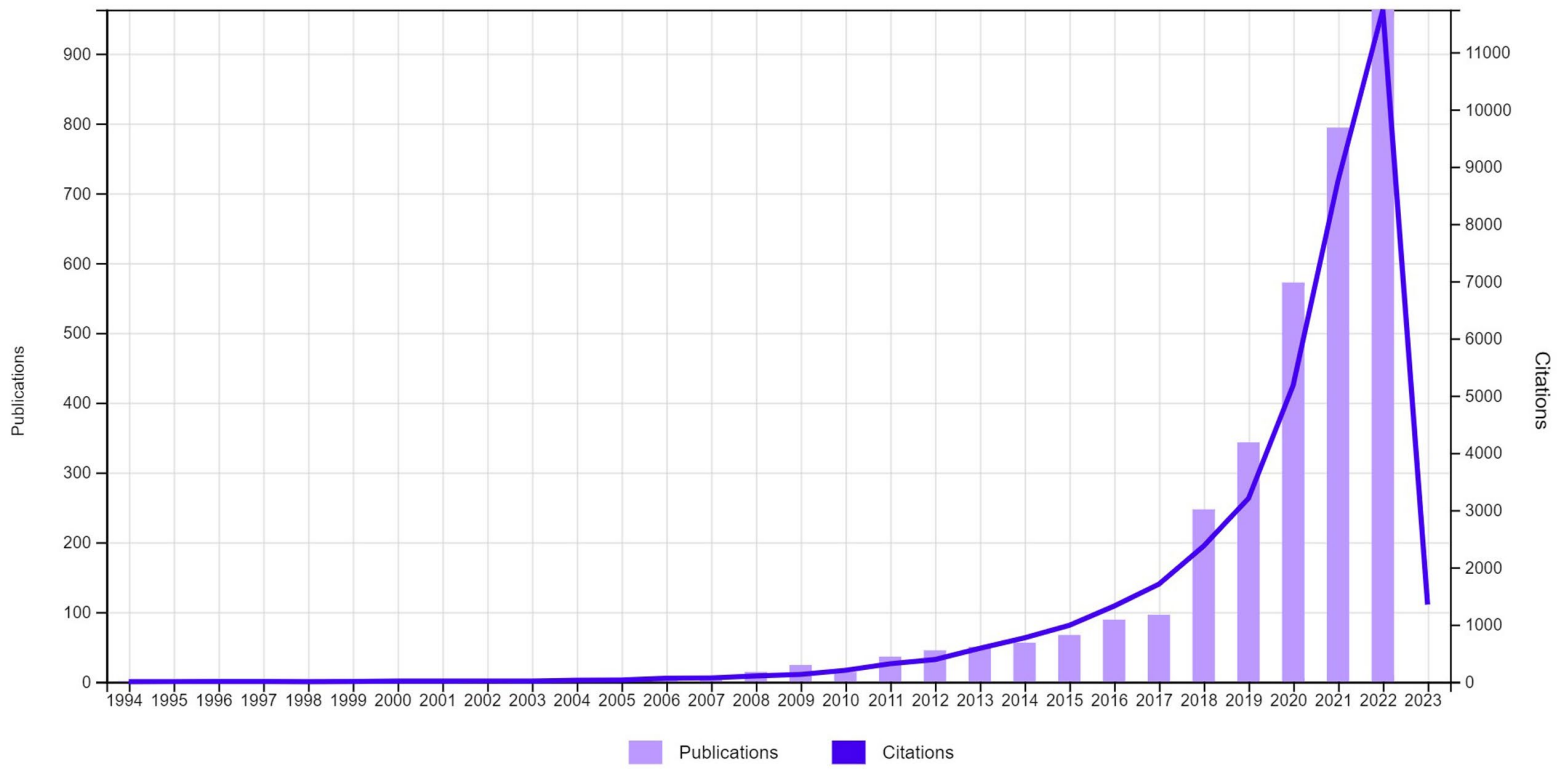
Publication and citation trends for articles on digital literacy based on web of science (Retrieved April 8, 2023).

From 3,433 articles on DL, education dominates (39.4%), followed by communication (8.97%) and information sciences (8.09%). Research began in 1994, with fewer than 100 publications annually until 2017 (Figure 1). A significant increase in publications occurred from 2017 (*n* = 96) to 2018 (*n* = 247), a 2.5x increase. Citations also significantly increased from 5,188 in 2020 to 8,772 in 2021.

**Figure 2:**
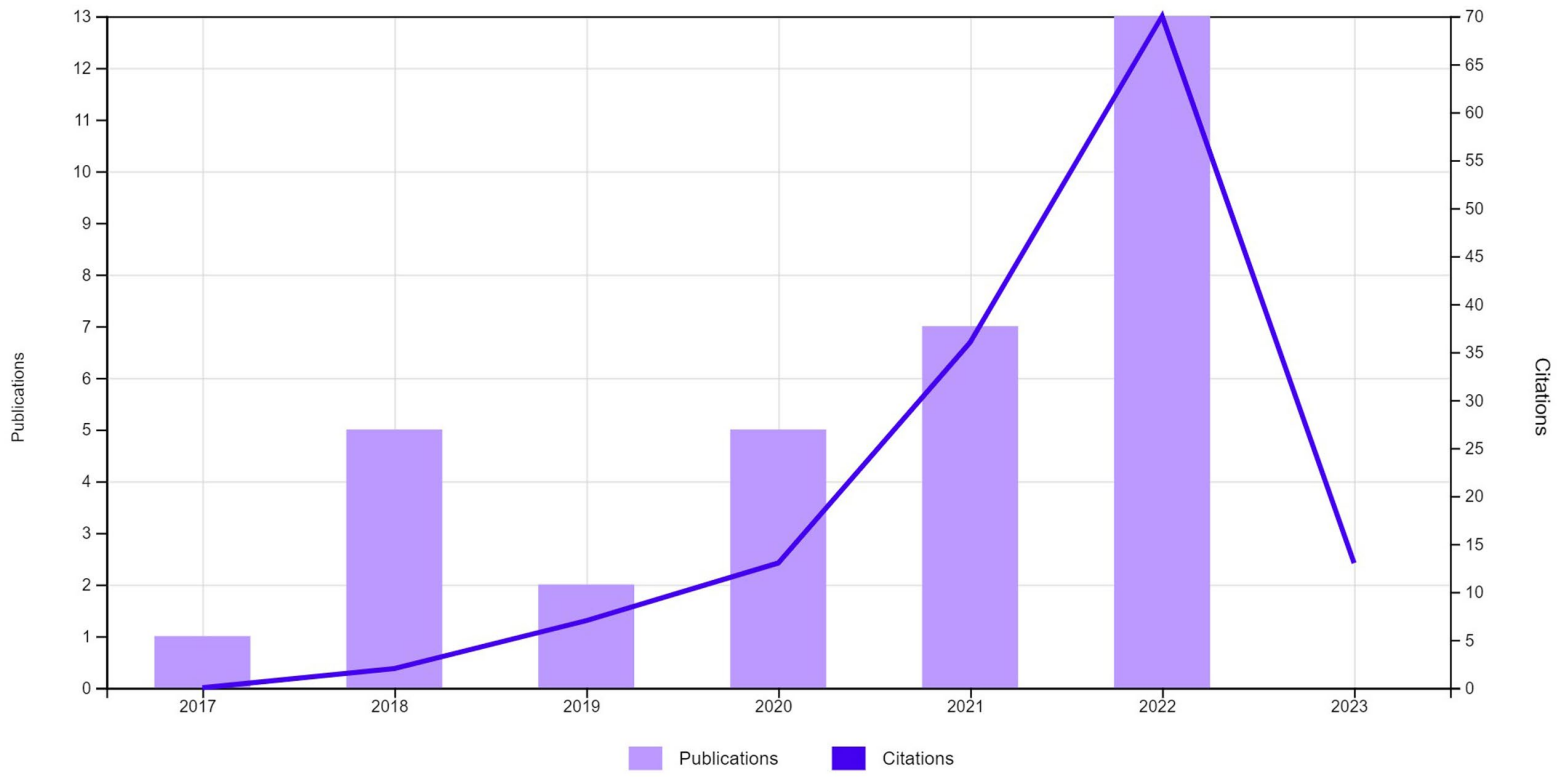
Publication and citation trends for articles on digital literacy and employability based on web of science (Retrieved April 8, 2023).

From 33 articles on DL and employability, education dominates (45.46%), followed by environmental studies (15.15%) and green sustainable science (9.9%). Research began in 2017 with one publication (Figure 2). A significant increase occurred in 2018 with five publications, an increase of 5x from the previous year. Citations also significantly increased from 13 in 2020 to over 36 in 2021.

**Figure 3:**
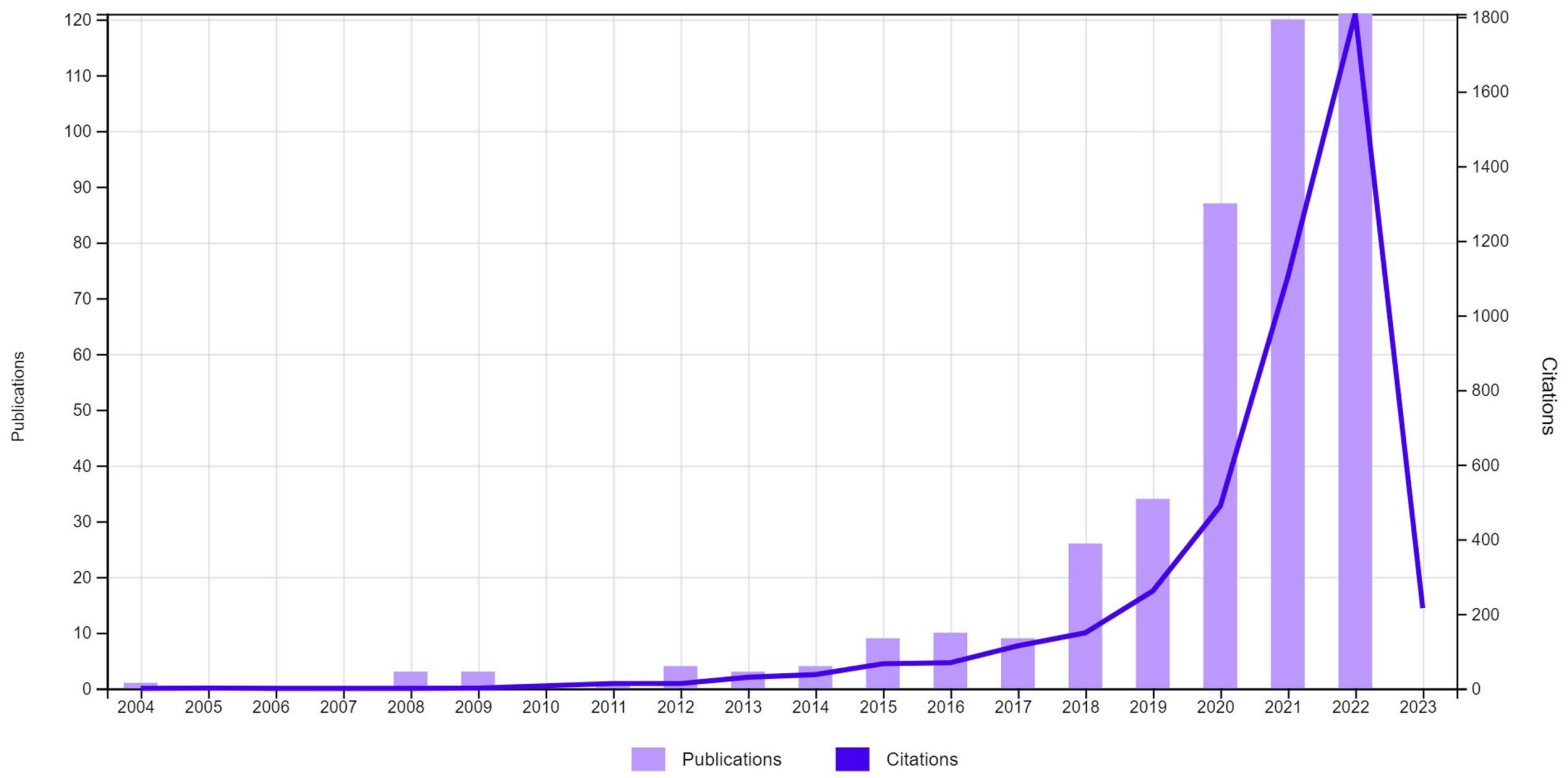
Publication and citation trends for articles on digital literacy and innovative work behavior (Retrieved April 8, 2023).

From 435 articles on DL and IWB, education was the dominant category (30.80%), followed by environmental sciences (8.5%) and communication (7.59%). Research began in 2004, with nine publications by 2017 (Figure 3). A significant increase occurred in 2018 with 26 publications, an increase of 2.88x from the previous year. Citations also significantly increased from 487 in 2020 to over 1,106 in 2021.

#### 2.2.2 Science mapping analysis

**Figure 4:**
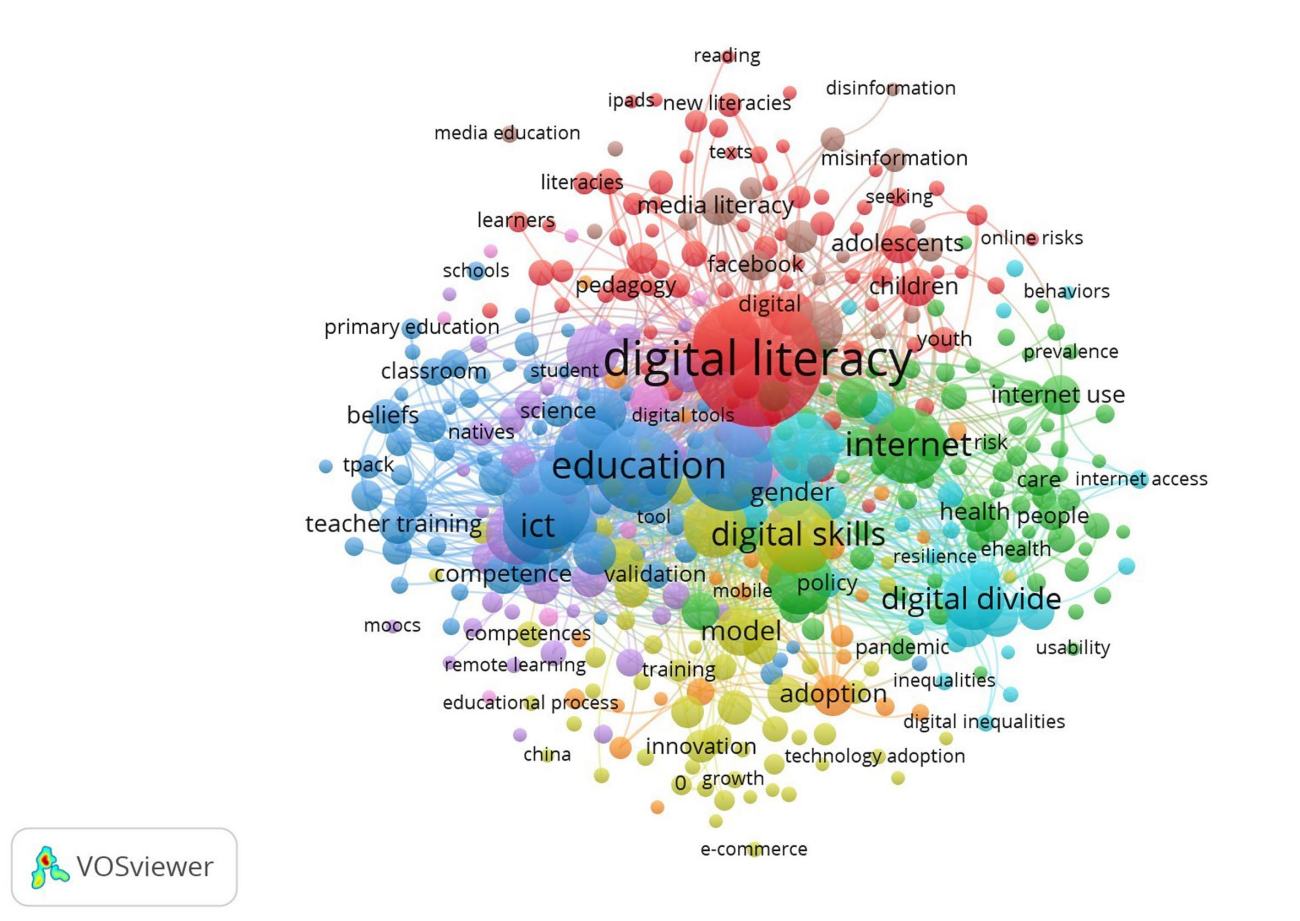
Co-occurrence network visualization of digital literacy.

We constructed a science map for the topics of DL, employability, and IWB. In 3,433 articles on DL, we found 9,731 keywords, 411 thresholds, and nine thematic clusters (Figure 4). Cluster 1 focuses on digital literacy (red nodes, 78 keywords), Cluster 2 focuses on health (green, 76 keywords), Cluster 3 focuses on education (blue, 68 keywords), Cluster 4 focuses on digital technology (yellow, 62 keywords), Cluster 5 focuses on learning systems (violet, 42 items), Cluster 6 focuses on digital divide (light blue, 30 keywords), Cluster 7 focuses on technology acceptance (orange, 26 keywords), Cluster 8 focuses on social media and information credibility (brown-red, 20 keywords), and Cluster 9 focuses on education and gender disparities regarding computer use (pink, 9 keywords).

**Figure 5:**
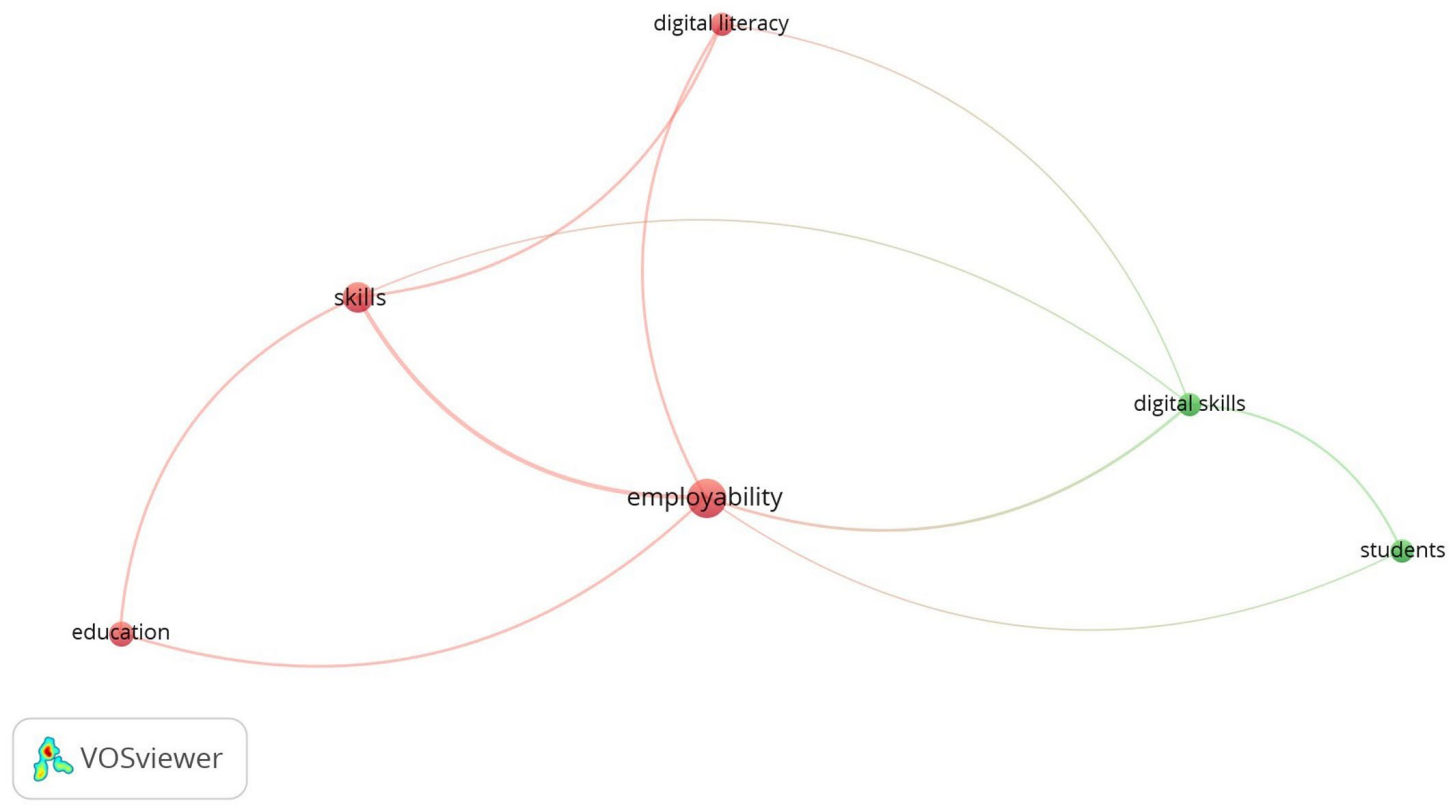
Co-occurrence network visualization of digital literacy and employability.

In 33 articles on DL and employability, we found 192 keywords, 6 thresholds, and 2 clusters (Figure 5). Cluster 1 focuses on education (4 keywords), and Cluster 2 focuses on digital skills and students (2 keywords). In Cluster 1, DL relates to employability, and for Cluster 2, we found no relation with employability.

**Figure 6:**
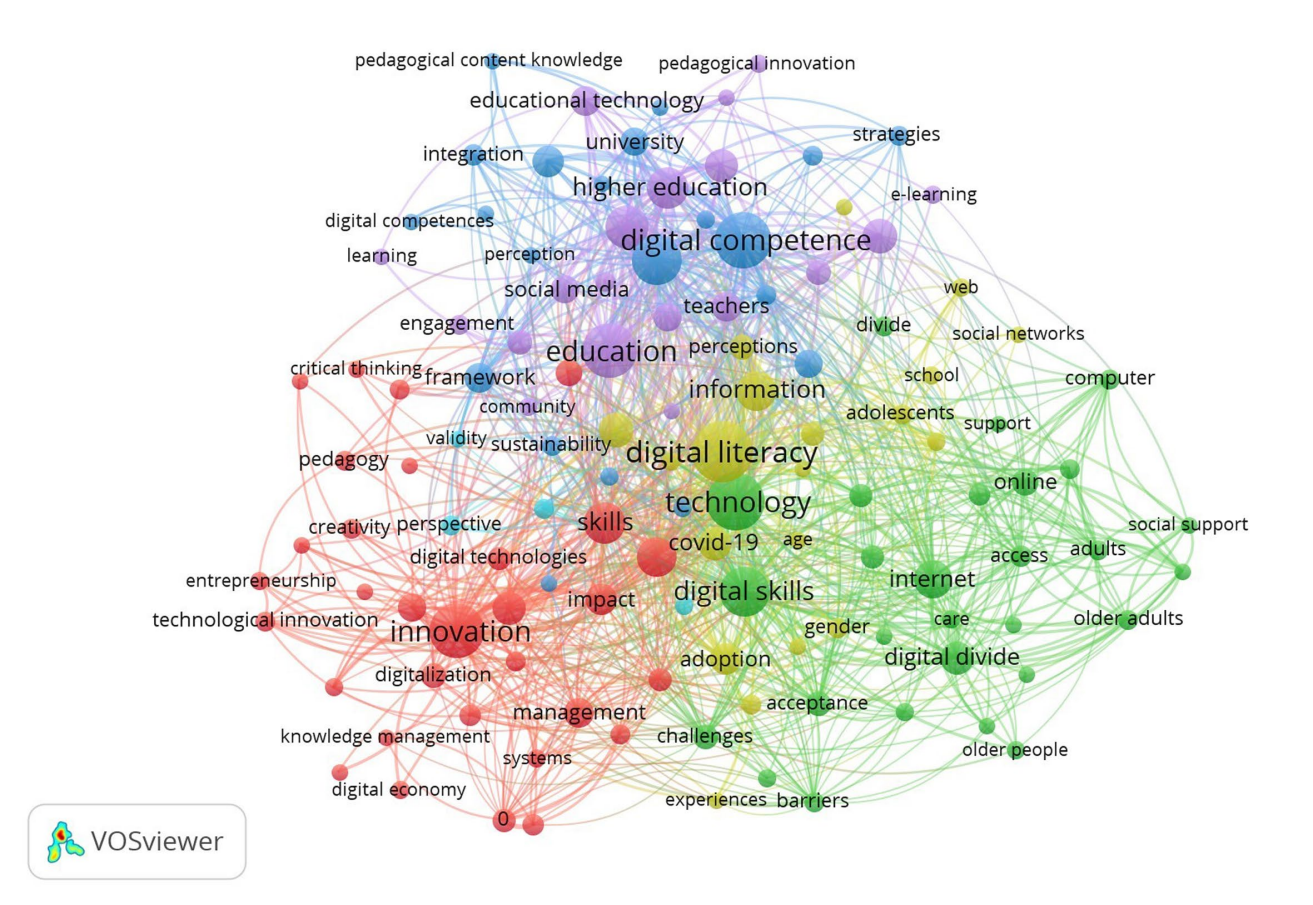
Co-occurrence network visualization of digital literacy and innovative work behavior.

From 435 articles on DL and IWB, we found 2,107 keywords, 123 thresholds, and 6 clusters (Figure 6). Cluster 1 focuses on innovation and management (31 keywords). Cluster 2 focuses on technology acceptance and the digital divide (28 keywords). Cluster 3 focuses on knowledge and skills (21 keywords). Cluster 4 focuses on digital technology (20 keywords). Cluster 5 focuses on education (19 keywords). Cluster 6 focuses on methodology (4 keywords).

#### 2.2.3 Summary

We found two major thematic clusters: education and communication. Limited studies, however, focus on business contexts. Research on DL, DL and employability, and DL and IWB peaked in 2018, with a second peak in 2020. We observed an increase in citations on combinations of the two topics during 2021, indicating a trend toward the topics, with DL largely associating with employability and IWB, and the two topics are expected to be studied more in the future. Increased research on these two topics in 2020 was likely prompted by the COVID-19 pandemic, which triggered the use of digital technology in organizations among employees exposed to it, especially when the use of technology was more complex and increasing daily.

## 3 Main study: systematic literature review

### 3.1 Methods

**Figure 7:**
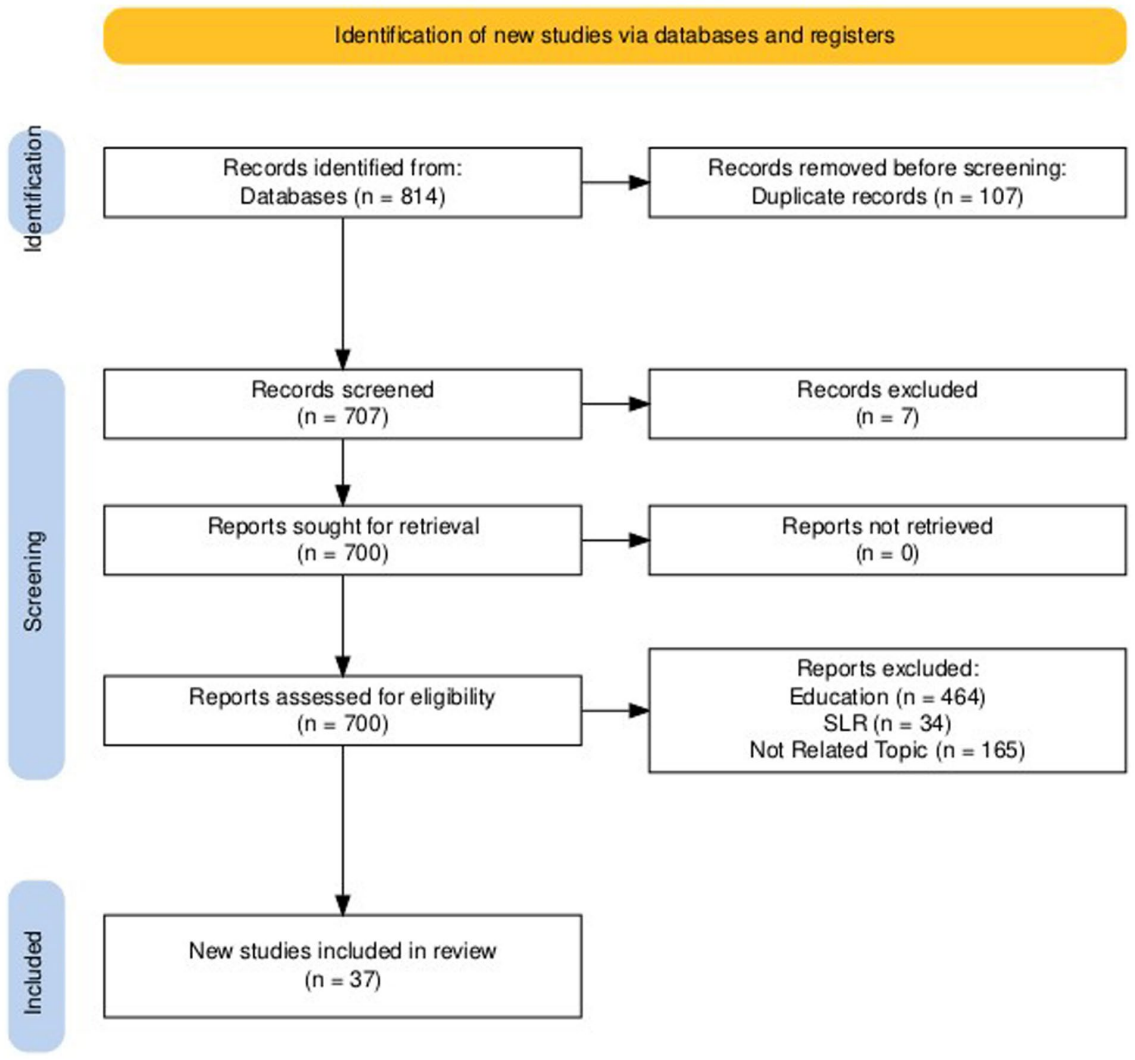
PRISMA flowchart.

We conducted this systematic literature review (SLR) for articles that were published until July 2024, using the PRISMA method to select articles (Figure 7). SLR can be explained as a systematic method for collecting, selecting, identifying, evaluating, and synthesizing the evidence from previous research to identify research gap and provide a research agenda (Tranfield et al., 2003). We expanded our database search beyond WoS to include SCOPUS, Academic Search Premier (EBSCO), and ProQuest to obtain a broader range of articles. We used similar Boolean operators with the bibliometric analysis; however, we excluded the term innovation during the SLR, which is broader and sometimes irrelevant to the research question about IWB (see Table 2). After removing duplicates and applying exclusion criteria, our final dataset comprised 37 articles.

**Table 2:** Inclusion criteria for systematic literature review.

			Reason
Database	Web of Science, SCOPUS, EBSCO (Academic Search Premier), and ProQuest		Web of Science and SOCPUS are the largest, so we could get all articles already published
Topic	Digital literacy and employability	Digital literacy and innovative work behavior	Corresponded with the research question to assess the relationship between digital literacy and both employability and innovative work behavior
Search string	(“digital literacy” OR “digital skills” OR “digital competence” OR “digital fluency”) AND (“employability” OR “perceived employability”)	(“digital literacy” OR “digital skills” OR “digital competence” OR “digital fluency”) AND (“innovative (work) behavio*” OR “idea generation” OR “idea promotion” OR “idea implementation” OR “idea creation” OR “idea realization”)	
Search Limitation	Journal articles, academic journals, and articles Peer reviewed Published up to July 23, 2024 English		To discover trends of recent articles Ensure that articles are sufficiently credible for analysis Ensure consistent analyses and predict trends for 2024 onward Clear investigation
Content Limitation	Empirical research (quantitative or qualitative) Non-education		Understand the novelty for further research Many articles in educational contexts, but limited to business contexts

### 3.2 Research trends

Table 3 demonstrates that the research on the two topics began in 2009, followed by studies in 2015 and 2018, and consistently from 2019 to 2022, with an average of 3 to 4 articles per year. However, in 2023, a notable surge occurred, with the number of articles reaching 12. This upward trend persisted, and as of mid-2024, there were already seven articles on the topic. We now first discuss the research approach, methods, and data analysis, and second the target group, industries, and countries.

**Table 3:** Result of systematic literature review.

**ID**	**Author**	**Title**	**Year**	**Variables**	**Continents**	**Countries**	**Industries/Target Respondents**	**Methods**	**Projects**	**Coaching/Training**
1	Lee et al.	Training older workers for technology-based employment	2008	DL=EM	North America	The United States	Unemployed adults	Mixed Method		Training
2	Woodley et al.	Technology mentors: enablers of ICT uptake in Australian small business	2015	DL=EM	Australia	Australia	SME	Qualitative	Project	The Enabled Tradie project
3	Bode and Gold	Adult training in the digital age	2018	DL=EM		G20		Qualitative	Project	Adult training programs
4	Lissitsa and Chachashvili-Bolotin	The effect of digital variables on perceived employability in an ethnic minority and the hegemonic group	2019	DL = EM	Asia	Israel	Random	Quantitative		
5	Palmeiro et al.	Digital inclusion programs: the case of the Basque Country	2019	DL=EM	Europe	the Basque	Country	Qualitative	Project	The KZgunea Program
6	Santoso et al.	The role of digital literacy in supporting performance through innovative work behavior: the case of Indonesia’s telecommunications industry	2019	DL=IWB	Asia	Indonesia	Telecommunications	Quantitative		
7	Santoso et al.	The role of creative self-efficacy, transformational leadership, and digital literacy in supporting performance through innovative work behavior: evidence from telecommunications industry	2019	DL=IWB	Asia	Indonesia	Telecommunications	Quantitative		
8	Bejaković and Mrnjavac	The importance of digital literacy on the labor market	2020	DL = EM	Europe	EU	Workers	Quantitative		
9	Guenther et al.	Digital inclusion in Central Australia: what is it and what makes it different?	2020	DL=EM	Australia	Australia	Country	Qualitative	Project	inDigiMOB
10	Guitert et al.	Basic digital competences for unemployed citizens: conceptual framework and training model	2020	DL=EM	Europe	Unknown (EU context)		Qualitative - DBR - ADDIE framework	Project	The SELFEE Project
11	Atsiyeva et al.	Problems of agency work during the coronavirus crisis: a case of Kazakhstan	2021	DL = EM	Asia	Kazakhstan	Employees and employers	Quantitative		
12	Holmes and Burgess	Homelessness prevention through one-to-one coaching: the relationship between coaching, class stigma, and self-esteem	2021	DL=EM	Europe	The United Kingdom		Qualitative	Project	Coaching: New Horizons programme
13	Pilav-Velić et al.	Digital or innovative: understanding “digital literacy–practice–innovative work behavior” chain	2021	DL = IWB	Europe	Bosnia and Herzegovina	Pharmaceuticals	Quantitative		
14	Pinto and Cardoso	Facebook as a local and community digital media? Experiences impacting on the unemployed audiences of the project “REviver na Rede”	2021	DL=EM	Europe	Portugal		Qualitative	Project	REviver na Rede
15	Erhan et al.	From conventional to digital leadership: exploring digitalization of leadership and innovative work behavior	2022	DL = IWB	Asia and Europe	Turkey	Textiles	Quantitative		
16	Herrero-De-La-Fuente et al.	Digital skills and technological accessibility as challenges for the labor market insertion of people with disabilities in the audiovisual sector	2022	DL=EM	Europe	Spain	Audio-Visual	Qualitative		Training
17	Kaki et al.	Skills mismatch in the agricultural labor market in Benin: vertical and horizontal mismatch	2022	DL=EM	Africa	Benin	Agriculture	Quantitative		
18	Pavić-Rogošić et al.	Digitalna.hr project—ideas, implementation and activities for integrating vulnerable groups into the digital society	2022	DL=EM	Europe	Croatia		Qualitative	Project	Digitalna.hr project
19	Akhmadi and Tsakalerou	Exploring gender imbalances in innovation and entrepreneurship: evidence from a global south country	2023	DL == IWB	Asia	Kazakhstan	Manufacturing, construction, oil & gas sector	Quantitative		
20	Alao and Brink	Information and communication technology management for sustainable youth employability in underserved society: technology use for skills development of youths	2023	DL=EM	Africa	South Africa	Youth	Quantitative		
21	De Marco et al.	Jobless and burnt out: digital inequality and online access to the labor market	2023	DL=EM	Europe	Spain	Job Seeker	Quantitative		
22	Kortmann et al.	Digitalization in occupations and self-perceptions of aging of older workers	2023	DL=EM	Europe	Germany	Older Workers	Quantitative		
23	Lakomý	Effects of digital skills and other individual factors on retirement decision-making and their gender differences	2023	DL=EM	Europe	European Countries		Quantitative		
24	Plummer	Multidimensions of digital inequality of the TANF population	2023	DL=EM	North America	The United States		Quantitative	Project	TANF
25	Riaz et al.	An augmentation for innovation: psycho-tech innovative work behavior model through an intellectual risk-taking pathway	2023	DL=IWB	Asia	Pakistan	Health and Education	Quantitative		
26	Rîndașu et al.	Digitalisation and skills adequacy as determinants of innovation for sustainable development in EU countries: A PLS-SEM Approach	2023	DL=EM	Europe	27 European Countries		Quantitative		
27	Spurava and Kotilainen	Digital literacy as a pathway to professional development in the algorithm-driven world	2023	DL=EM	Europe	Finland & Latvia		Qualitative	Project	European ySKILLS
28	Taylor et al.	Physical to virtual: challenges and opportunities for a neighborhood-based employment support initiative	2023	DL=EM	Europe	The United Kingdom		Qualitative	Project	neighborhood-based employment support program
29	Wang et al.	Digital revolution and employment choice of rural labor force: evidence from the perspective of digital skills	2023	DL=EM	Asia	China	Household	Quantitative		
30	Yende	Importance of digital skills to South African opera artists to improve their employability	2023	DL=EM	Africa	South Africa	Opera Firms and Actors	Qualitative		
31	Arion et al.	Determining digitalization issues (ICT adoption, digital literacy, and the digital divide) in rural areas by using sample surveys: the case of Armenia	2024	DL=EM	Asia	Armenia	Rural households	Quantitative		
32	Audrin et al.	Digital skills at work–conceptual development and empirical validation of a measurement scale	2024	DL=EM	North America	The United States	Amazon Mechanical Tuck USA	Mixed-Method		
33	Gao and Gao	How does digital leadership foster employee innovative behavior: a cognitive–affective processing system perspective	2024	DL == IWB	Asia	China	Manufacturing	Quantitative		
34	Kerdsawad and Lekcharoen	The development of digital competencies: for Royal Thai Armed Forces Headquarters Lead to an Intelligent Headquarters	2024	DL=EM	Asia	Thailand	Armed Forces	Mixed-Method		
35	Lou et al.	Assessing the role of HRM and HRD in enhancing sustainable job performance and innovative work behaviors through digital transformation in ICT companies	2024	DL == IWB	Asia	China	ICT Companies	Quantitative		
36	Weerasombat et al.	Skill redefinition and classification, capitalism, and labor process theory of work: evidence from Thailand	2024	DL=EM	Asia	Thailand	Multi-industry	Mixed-Method		
37	Zhang et al.	What are the digital skills sought by scientific employers in potential candidates?	2024	DL=EM	North America	The United States	Scientists	Quantitative		

#### 3.2.1 Research approach—methods and data analysis

Approximately 32% of the articles used qualitative methods, 57% were quantitative, and the remaining 11% were mixed-methods studies. The qualitative studies used design-based approaches (Guitert et al., 2020), realist-informed formative approaches (Guenther et al., 2020) and descriptive phenomenology approaches (Yende, 2023). Data collection in the qualitative studies varied and included in-depth interviews (Herrero-De-La-Fuente et al., 2022; Holmes and Burgess, 2021) and surveys and questionnaires (Pinto and Cardoso, 2021; Woodley et al., 2015; Kerdsawad and Lekcharoen, 2024). Other qualitative studies used examinations, evaluations, and reporting of projects (Bode and Gold, 2018; Palmeiro et al., 2019; Pavić-Rogošić et al., 2022). The qualitative studies commonly focused on specific projects (Plummer, 2023; Taylor et al., 2023), rather than generalizing to broader contexts. The quantitative studies used explanatory surveys (Arion et al., 2024; Erhan et al., 2022; Kaki et al., 2022; Pilav-Velić et al., 2021; Santoso et al., 2019a; Santoso et al., 2019b), questionnaires with open-ended questions (Atsiyeva et al., 2021), or questionnaires with closed-ended questions (Akhmadi and Tsakalerou, 2023). Some studies used secondary data (Bejaković and Mrnjavac, 2020; Lissitsa and Chachashvili-Bolotin, 2019), while Zhang et al. (2024) sourced data directly from online platforms. For data analysis, several software packages were used, including NVivo during qualitative analysis, SPSS during quantitative analysis (Lou et al., 2024; Erhan et al., 2022; Pilav-Velić et al., 2021), LISREL (Santoso et al., 2019a; Santoso et al., 2019b), AMOS (Lou et al., 2024; Erhan et al., 2022; Pilav-Velić et al., 2021), SMART PLS 4 (Gao and Gao, 2024; Riaz et al., 2023; Rîndașu et al., 2023), STATA (Lakomý, 2023; Kortmann et al., 2023), Ms. Excel (Alao and Brink, 2023), R (Audrin et al., 2024; De Marco et al., 2023), and MPlus (Kortmann et al., 2023).

#### 3.2.2 Target group—sectors and countries

Twelve qualitative research articles examined the importance of DL across diverse populations and target groups, including small business owners (Woodley et al., 2015), homeless people (Holmes and Burgess, 2021), households (Wang et al., 2023), vulnerable groups (Herrero-De-La-Fuente et al., 2022; Pavić-Rogošić et al., 2022), older adults (Bode and Gold, 2018; Palmeiro et al., 2019), and unemployed people (Guitert et al., 2020; Palmeiro et al., 2019; Pinto and Cardoso, 2021). Many quantitative studies focused on employees across industries, such as textiles, pharmaceuticals, telecommunications, and agriculture. Thirty-two percent of the articles assessed developing countries, and the remaining 54% focused on developed countries. The remaining 14% encompassed both countries or could be categorized accordingly.

Approximately 43% examined subjects in OECD countries, 14% included both OECD and non-OECD countries, and the remaining 43% concentrated on non-OECD countries. Some quantitative studies explored the relationship between DL and other factors at country or continent levels (Bejaković and Mrnjavac, 2020).

#### 3.2.3 Emerging topic of digital literacy

No study explicitly investigated the interplay between DL, employability, and IWB. A total of 29 studies explored the relationship between DL and employability, and eight concentrated on DL and IWB. Some articles cited Martin (2005) and Ferrari (2012) to explain DL. Martin (2005) was cited by Pilav-Velić et al. (2021), Pinto and Cardoso (2021), Santoso et al. (2019a), and Santoso et al. (2019b), and Ferrari (2012, 2013) by Bejaković and Mrnjavac (2020), Pilav-Velić et al. (2021), Guitert et al. (2020), Palmeiro et al. (2019), and Audrin et al. (2024). Ferrari (2012) also refers to Martin (2005), suggesting that understanding DL requires digital competency, particularly in Europe. The more recent articles often used Vuorikari et al. (2016) or Vuorikari et al. (2022), with an emphasis on DigComp also used in much research, such as Riaz et al. (2023), Spurava and Kotilainen (2023), Audrin et al. (2024), and Guitert et al. (2020).

DL received significant attention, especially after the European Commission released The European Digital Competence Framework 2018 (DigComp), which provides a comprehensive understanding of digital competencies. The DigComp framework has become a common reference in numerous digital competence studies. Digital competence involves confident, critical, and responsible use of and engagement with digital technologies for learning at work and participation in society. Digital competencies include Ferrari’s (2012) seven dimensions of information management, collaboration, communication and sharing, creation of content and knowledge, ethics and responsibility, evaluation and problem-solving, and technical operations. The digital competence framework continued to develop, even into 2022 called DigComp 2.2 with 5 dimensions such as Information and data literacy, Communication and collaboration, Digital content creation, Safety, and Problem-solving (Vuorikari et al., 2022).

### 3.3 Measurement

There is a lack of consistency in measuring DL. Since 2023, no consensus has been reached on DL measurement indicators, whether for individuals, organizations, or companies. A variety of measures have been used to assess DL; Van Laar et al. (2017); Van Deursen et al. (2016), Eurostat, based on DigComp 2.0 (European Commission, Directorate-General for Employment, Social Affairs and Inclusion, 2018; Vuorikari et al., 2016), DigComp 2.2, Israel’s Central Bureau of Statistics 2010 (Lissitsa and Chachashvili-Bolotin, 2019), China Family Panel Studies (CFPS) - access level and use levels, SHARE dataset about digital skills and demand for digital skills at work, The GEAS Dataset about digitalization level and change in digital literacy and Ng (2012) are used as sources in measuring DL. Van Laar et al. (2017) discuss four dimensions of DL, including technical aspects, information management, critical thinking, and problem-solving. Bejaković and Mrnjavac (2020) use four areas of digital competencies, including information, communication, problem-solving, and software skills. Ng (2012) uses three dimensions—technical, cognitive, and social emotion—which comprise 10 indicators. Measurement of employability relies on Rothwell and Arnold (2007) for perceived employability (Lissitsa and Chachashvili-Bolotin, 2019).

De Jong and Den Hartog (2010) developed indicators for IWB, which are used in four articles (Riaz et al., 2023; Erhan et al., 2022; Santoso et al., 2019a; Santoso et al., 2019b). De Jong and Den Hartog’s (2010) scale consists of four dimensions—idea exploration, generation, championing, and implementation—comprising 10 items. Other researchers have used different IWB scales. Pilav-Velić et al. (2021) used a 13-item scale from Zhou and George (2001), while Lou et al. (2024) combined scales from Scott and Bruce (1994) and Kleysen and Street (2001). Gao and Gao (2024) relied solely on Zhu and Zhang’s (2020) scale.

### 3.4 Variables in the conceptual model

DL appears to serve as an antecedent, mediator, and moderator in the studies examined. Conversely, employability is used mostly as an outcome, indicating its dependence on other factors. IWB was found to function as both a mediator and outcome. DL is associated with various outcomes, including perceived employability (Lissitsa and Chachashvili-Bolotin, 2019), employment (Bejaković and Mrnjavac, 2020; Wang et al., 2023), online job-seeking skills (De Marco et al., 2023), digital practices, attitudes toward digitalized innovation, and IWB (Pilav-Velić et al., 2021; Riaz et al., 2023). It has a direct effect on perceived employability (Lissitsa and Chachashvili-Bolotin, 2019), employment (Bejaković and Mrnjavac, 2020), online job-seeking skills (De Marco et al., 2023), IWB (Riaz et al., 2023) and digital practices; and it has indirect effects on attitudes toward digitalized innovation. DL also has an indirect effect on IWB when considering mechanism variables such as digital practices and attitudes toward digitalized innovation (Pilav-Velić et al., 2021). However, limited research examines DL’s antecedents about these topics, with digital resources identified as a potential factor (De Marco et al., 2023).

**Figure 8:**
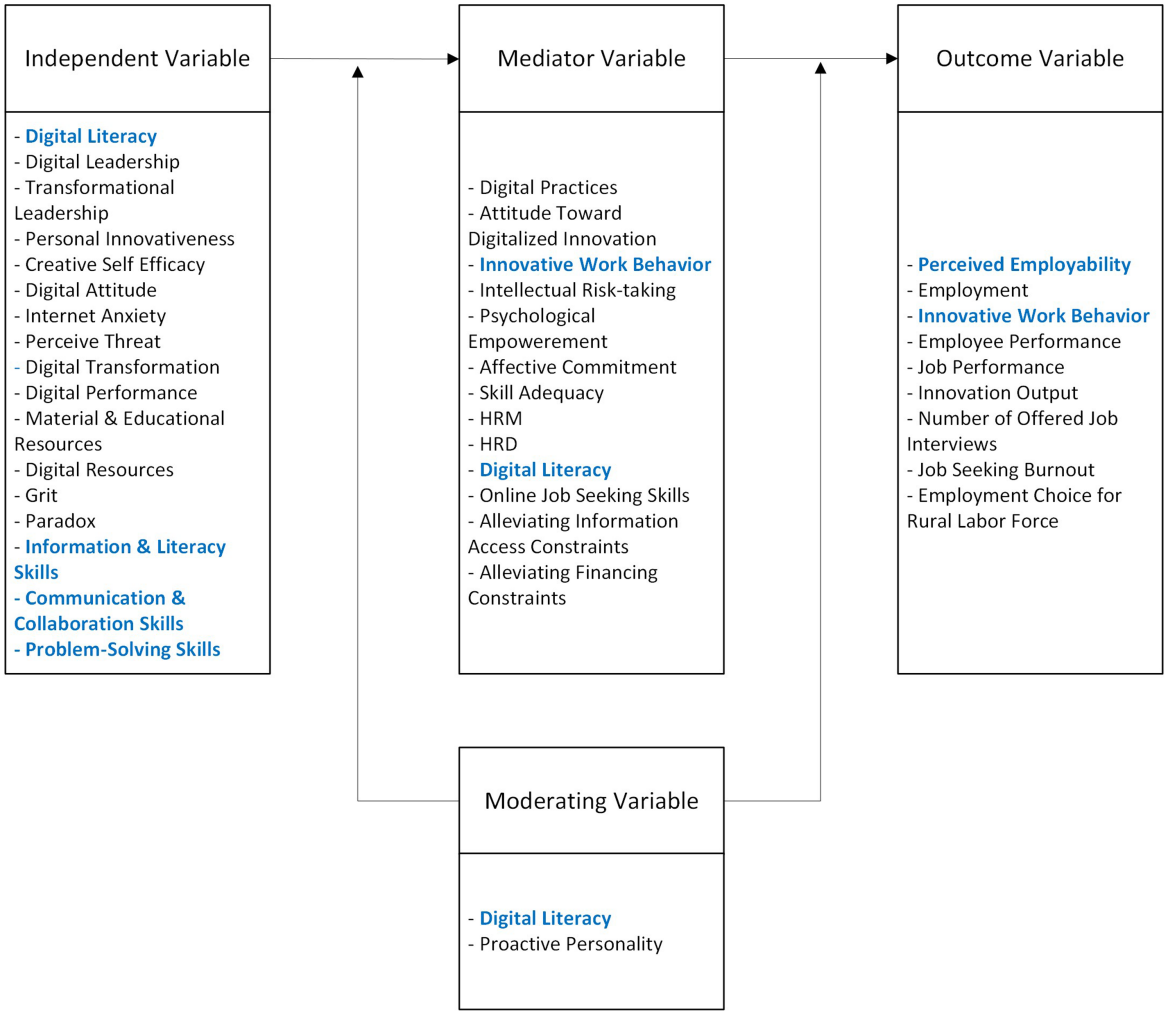
The conceptual relationship among digital literacy, employability, and innovative work behavior.

For IWB, antecedents such as digital leadership (Erhan et al., 2022; Gao and Gao, 2024), DL (Pilav-Velić et al., 2021; Riaz et al., 2023), digital transformation (Lou et al., 2024), personal innovativeness (Pilav-Velić et al., 2021), transformational leadership (Santoso et al., 2019a; Santoso et al., 2019b) and creative self-efficacy (Santoso et al., 2019b) have been identified. Both digital leadership and transformational leadership influence IWB, and outcomes linked to such behavior commonly assess employees’ performance (Santoso et al., 2019a; Santoso et al., 2019b). DL represents a mechanism variable, acting as a moderator in the relationship between IWB and employee performance (Santoso et al., 2019a; Santoso et al., 2019b). Digital skills are antecedents for employability (Lissitsa and Chachashvili-Bolotin, 2019), although there are few quantitative studies assessing the relationship between DL and employability, particularly in conceptual models (see Figure 8).

### 3.5 Theoretical frameworks with the constructs

Several theoretical frameworks suggest links between DL and employability. Human capital theory (Becker, 2009) emphasizes the importance of improving employees’ skills, knowledge, and abilities to increase the stock of human capital in organizations, particularly among skill mismatches in the labor market. The theory suggests that digital skills enhance employability (Kaki et al., 2022; Lissitsa and Chachashvili-Bolotin, 2019). Job competition theory highlights skills that job markets demand, motivating people to enhance their skills to secure higher-level positions (Devereux, 2002). Sicherman and Galor’s (1990) career mobility theory describes how skills integrate into career paths, with higher-skilled people enjoying better occupational opportunities. Sattinger’s (1993) assignment theory suggests that employees’ skills determine wages and career characteristics. Social capital theory elucidates how online community activities enhance employment prospects and employability (Habets et al., 2021). The technology adoption model suggests that attitudes toward technology influence access and use of both information and communication technology and hence that the use of technology increases internet anxiety and perceived threats, potentially influencing employability (Lissitsa and Chachashvili-Bolotin, 2019). Gestalt theory, which focuses on perceptions and holistic interpretations of the world, has been used to investigate the relationship between DL and employability (Mungan, 2021; Pinto and Cardoso, 2021).

The labor process theory (LPT) advocates for a re-evaluation of essential worker skills in light of the evolving workplace landscape. As traditional skillsets may become obsolete due to technological advancements and shifting market demands, LPT offers a framework for redefining and identifying the critical competencies necessary for success in contemporary and future labor markets (Weerasombat et al., 2024). The sustainability livelihood theory provides a valuable framework for examining how human and environmental attributes, such as human capital, financial capital, social capital, physical capital, and natural capital, can influence the sustainability of employment facilitated by ICT (Alao and Brink, 2023). Substitution Theory posits that technological advancements can lead to a decline in labor demand as machines and software can perform tasks traditionally done by humans more efficiently and cost-effectively. In the context of the digital economy, this theory is particularly relevant due to the rapid pace of technological innovation and automation (Wang et al., 2023). The theory of feasibility ability posits that an individual’s capacity to participate effectively in a particular domain, such as employment, depends on their possession of the necessary abilities or skills (e.g., digital skills) (Wang et al., 2023). According to resource-based theory, digital skills (DS) play a crucial role for rural laborers to identify opportunities and integrate resources effectively (Wang et al., 2023).

Five theoretical frameworks have also been used to explain the relationship between DL and IWB. Self-determination theory, with its emphasis on intrinsic motivation and autonomy, can help explain how individuals’ perceptions of digital literacy can influence their engagement in innovative activities. The theory of planned behavior, on the other hand, can shed light on the role of social norms, perceived behavioral control, and attitudes in shaping individuals’ intentions and behaviors of innovation (Riaz et al., 2023). Innovation diffusion theory shows that individuals’ characteristics such as interpersonal, social skills, and digital skills can contribute to the speed of adopting and creating innovation and technology (Rîndașu et al., 2023). The upper echelons theory and the leadership theory explain how leaders’ digital competencies influence employees and organizations (Erhan et al., 2022; Gao and Gao, 2024).

### 3.6 Research on digital literacy and employability

Extant research on DL and employability consists of four mixed-method, twelve qualitative, and thirteen quantitative studies. Such research began in 2008 (Lee et al., 2008) and continued during subsequent years, with studies conducted in 2015 (Woodley et al., 2015), 2018 (Bode and Gold, 2018), 2019 (Lissitsa and Chachashvili-Bolotin, 2019; Palmeiro et al., 2019), 2020 (Bejaković and Mrnjavac, 2020; Guenther et al., 2020; Guitert et al., 2020), 2021 (Atsiyeva et al., 2021; Holmes and Burgess, 2021; Pinto and Cardoso, 2021), 2022 (Herrero-De-La-Fuente et al., 2022; Kaki et al., 2022; Pavić-Rogošić et al., 2022); 2023 (Alao and Brink, 2023; De Marco et al., 2023; Kortmann et al., 2023; Lakomý, 2023; Plummer, 2023; Rîndașu et al., 2023; Spurava and Kotilainen, 2023; Taylor et al., 2023; Wang et al., 2023; Yende, 2023), and 2024 (Arion et al., 2024; Audrin et al., 2024; Kerdsawad and Lekcharoen, 2024; Weerasombat et al., 2024; Zhang et al., 2024). Most studies focus on OECD and developed countries, with seven addressing countries outside of OECD and developing nations.

Lee et al. (2008, p. 19) referred to DL as “attitudes toward computers or computer experience,” which is a narrower concept than DL. In that study, unemployment was found to be caused by a lack of skills, especially computer skills, and a lack of access to training. Unemployed people want to learn more about computers, but they also want to return to their jobs. Woodley et al. (2015, p. 659) similarly used the terms “ICT skills or ICT capabilities,” focusing on the ability to use specific software relevant to small and medium-sized enterprises (SMEs), finding that SME owners lack expertise with technology but need it to access information and help them run their businesses.

These studies offer two perspectives—DL for SME owners, and employability skills among ICT mentors. Bode and Gold (2018) assess skills that complement technology and that are necessary for adults who experience difficulties with obtaining employment (i.e., employability skills), suggesting that the G20 establish national training programs to improve digital skills and employability. Lissitsa and Chachashvili-Bolotin (2019) explore three digital aspects—digital skills, uses, and attitudes—using quantitative methods. Digital skills were measured in terms of the ability to use applications such as Google and email, with results suggesting that digital skills affect employability. Palmeiro et al. (2019) conducted a qualitative study on digital inclusion, finding that DL represents an instrument of digital inclusion with which countries can develop digital citizens and that helps with employability. Bejaković and Mrnjavac (2020) conducted a quantitative study on DL, suggesting that people with digital skills have higher employment rates than people without such skills. In contrast, Guitert et al. (2020) developed a conceptual framework of digital competencies that focuses on unemployment. Guenther et al. (2020) assess digital inclusion, finding that DL represents a fundamental human right. Atsiyeva et al. (2021) assess online employability, which adapts well when employees have significant DL. Pinto and Cardoso’s (2021) qualitative study assessed DL, and Holmes and Burgess (2021) assessed coaching for homelessness, which increases confidence and self-esteem through financial management, digital skills, and employability. Pavić-Rogošić et al.’s (2022) qualitative study on DL refers to DigComp 2.2, explaining that bridging the digital gap among vulnerable groups starts with an idea and moves to implementation and activities in the digitalna.hr project. Herrero-De-La-Fuente et al.’s (2022) qualitative study assessed digital skills for people with disabilities in the audio-visual industry, recommending accessibility to various technology tools. Kaki et al. (2022) quantitatively compare the digital skills of employees and employers’ perspectives, to identify skill gaps in agriculture.

Qualitative research is vital to identifying patterns and gaining in-depth insights into concepts. When assessing DL and employability, qualitative studies commonly focus on digital inclusion and the digital divide in the digital economy. Digital inclusion is frequently associated with DL across various studies, and such qualitative research illustrates interconnections among digital skills, particularly regarding technology use and their potential to improve employment capabilities while reducing society’s digital divide. Research on DL has evolved, exploring disparate aspects such as attitudes toward computer skills in 2008 (Lee et al., 2008), ICT skills in 2015 (Woodley et al., 2015), and DL in 2018 (Bode and Gold, 2018).

### 3.7 Research on digital literacy and innovative work behavior

Eight of the 37 articles discussing DL and IWB use quantitative designs which contrast with those discussing DL and employability. The development of DL led to the discovery of how digital transformation helps companies innovate digitally (Martin and Grudziecki, 2006). Research on this topic uses respondents from one company, so generalizability is narrower. Santoso et al. (2019a), and Santoso et al. (2019b) treat DL as a moderator in the relationship between IWB and employee performance, finding a significant result. Pilav-Velić et al. (2021) found an indirect relationship between DL and IWB, suggesting that DL influences such behavior through other mediators. Similarly, Erhan et al. (2022) and Gao and Gao (2024) report that digital leadership has positive influences on all dimensions of IWB, suggesting that leaders with DL skills foster and encourage such behavior among employees. Notably, five of the eight DL and IWB articles were conducted in developing and non-OECD countries.

### 3.8 Contextual approaches

To increase the relevance and usefulness of research results, contextual consideration can be used to ensure appropriate research methods and may lead to better questionnaire design and accurate data interpretation. Based on Whetten (2009), context can be categorized into two approaches, namely contextualizing theory and theorizing about context. These two approaches also focus on theory application and theory improvement. In addition, these approaches are simplified by Farndale et al. (2023) who focus on context-dependent theorizing from Whetten (2009) and variable-oriented theorizing.

Notable examples of variable-oriented theorizing emerge in the works of Atsiyeva et al. (2021), Lissitsa and Chachashvili-Bolotin (2019), Pilav-Velić et al. (2021), and Kaki et al. (2022). These studies use context variables, such as the generation of respondents (e.g., X generation, Y generation, baby boomers), cultural diversity, gender, education, and organizational tenure, as control variables in their research. Conversely, other articles adopt a context-dependent theorizing approach. For instance, Atsiyeva et al. (2021) and Erhan et al. (2022) delve into the impact of COVID-19 on workplaces and labor markets, although the focus is on conditions during the pandemic rather than a comparative analysis with pre-pandemic conditions. Guenther et al. (2020) focus on the digital landscape in Australia, examining factors influencing increased digital literacy. Woodley et al.'s (2015) research states three levels of digital literacy in Australia, distinct from digital competency in Europe. Palmeiro et al. (2019) concentrate on digital literacy in the Basque Country, and Pavić-Rogošić et al. (2022) investigate the implementation of digital society in Croatia. These studies show that the country where the research was conducted will influence the differences in digital literacy measurements. Apart from that, the digital level in the country will also influence the condition of digital literacy in society and the handling of different trainings.

Bode and Gold (2018), Bejaković and Mrnjavac (2020), and Pinto and Cardoso (2021) emphasize the importance of international guidelines and policies from organizations like the G20 and the European Union, offering countries, organizations, and individuals opportunities to develop their skills and competencies, one of which exists through career communities that can be easily connected via ICT. Guitert et al.'s (2020) research focuses on digital literacy for unemployed individuals, while Herrero-De-La-Fuente et al. (2022) work underscores the significance of tailored digital literacy training for people with disabilities. Lee et al.'s (2008) research delve into the context of unemployed adults, exploring their obstacles and challenges in returning to work.

The articles examined in this study collectively highlight the relevance of context to the discussed topics. Contexts encompass a broad spectrum, ranging from international organizational perspectives, international organizations (e.g., G20 and the European Union), and specific countries (e.g., UK, US, Spain) to targeted groups/respondents (e.g., older adults, individuals experiencing homelessness, small business owners), and even media technology. These diverse contexts offer valuable insights and perspectives on the relationships and influences related to these topics. However, not all the articles elaborate on this contextual paradigm, thus the relevance and usefulness of the topics are not yet discussed optimally.

## 4 Discussion

### 4.1 Future research

This research assesses DL, employability, and IWBs in organizational contexts, especially regarding research approaches, methods, analyses, target groups, industries, countries, construct measures in conceptual models, and theoretical frameworks. We identify a number of interrelated future research based on a previous review of extant studies (see Table 4). Extant studies commonly assess unemployed people and employees, but little research compares employees’ perspectives with those of employers, owners, and leaders. It is important to recognize that employees’ views and expectations are different from those of superiors and that DL, employability, and IWBs are self-driven and inherently motivated. Such variables allow employees to fulfill fundamental needs for autonomy, competence enhancement, and social connectedness, thereby promoting personal and professional development (Montani et al., 2022). By engaging in DL, employees gain the skills and knowledge required to navigate digital landscapes, which, in turn, enhances their employability by making them more adaptable and competitive in job markets. Fostering IWBs allows employees to generate and implement new ideas, contributing to their personal growth and the organization’s overall success. These self-driven variables satisfy employees’ basic psychological needs and encourage ongoing development (Ryan and Deci, 2000; Montani et al., 2022).

**Table 4:** Results avenues for future research.

Methodology	Level of analysis	Individual level, team level, and organizational level Compare the different perspectives among employee, supervisor/manager, and employers/business owner
	Country	Developing country
	Context	Business, industry
	Method	Mixed methods for digital literacy and employability topics Qualitative for digital literacy and innovative work behavior topics Mixed methods and others for interplay between digital literacy, employability, and innovative work behavior topics
Theoretical	Perspective	Using cross-cultural perspective to understand the different perspectives, assumptions, and needs of digital literacy in different nations and societies.
	Framework	Theory of planned behavior, technology acceptance model
	Measurement	Validation of a digital literacy measurement scale or tool for business context

Most studies focus on educational and social contexts, but few studies have been conducted in business and industry contexts, and therefore more research is needed. Countries outside of the OECD, particularly developing countries, commonly experience challenges due to a lack of a digital skills framework that adjusts to their contexts, which is compounded by the absence of guidance from organizations such as the Asian Commission and G20. DL should thus adapt to non-OECD countries and developing nations, which is crucial in diverse social, economic, and cultural contexts when developing frameworks that align with the unique needs and challenges of such countries. Adjustments should also consider available technological infrastructures, degrees of accessibility, financial constraints, and the education and training required to strengthen DL. For instance, the Indonesian national digital literacy program, launched in 2021, lagged 3 years behind the developed countries (Hani, 2021). Moreover, the program’s implementation was largely driven by the COVID-19 pandemic, highlighting the stark contrast with developed countries that had the luxury of pre-planning and robust IT infrastructure. This lack of preparedness exposed many Indonesian organizations, hindering their ability to adopt remote work models and digital tools effectively. By addressing such factors, policymakers and stakeholders can foster more inclusive and contextually appropriate approaches to enhancing digital skills and literacy in non-OECD and developing countries.

DL measures are diverse, resulting in a lack of consensus in the domain. Development and validation of DL measurement tools have been conducted primarily in social and educational contexts, leaving gaps in business and other contexts. The latest DL measure was released by the G20 in 2022, a pilot study of individuals and companies in Indonesia (Wang et al., 2022). The toolkit shows promise for future research, particularly in developing nations, non-OECD regions, and business contexts, though the initial toolkit was a one-off event and limited to a pilot study, underlining the need for extensive and diversified testing to ensure reliability and applicability. Such tests should include diverse industries, employees, and organizations so that the framework is improved and adaptable across contexts.

Employability measures traditionally fall into four categories—perceived future employability (Gunawan et al., 2021), perceived employability (Rothwell and Arnold, 2007), general employability (Van der Heijde and Van der Heijden, 2006), and graduate employability (Dacre Pool et al., 2014). When selecting a measure, future research should consider an organization’s context, with perceived future employability and graduate employability targeting students, and perceived employability and general employability targeting employees and employers.

Many researchers use IWB measures from either Janssen (2000) or De Jong and Den Hartog (2010), the latter of which adds the dimension of idea exploration, referring to the creative process of IWB that links with idea generation. We recommend De Jong and Den Hartog’s (2010) scale, which is the most recent instrument for assessing the construct. Studies commonly investigate the individual level, but a research gap exists at the group and organizational levels. Research should explore how the variables assessed in the current study manifest and interact in teams, in groups, and across organizational contexts. Assessing dynamics and implications at higher levels of analysis would provide valuable insights and enhance team performance and organizational outcomes.

Theoretical frameworks used in extant research commonly do not assess connections between technology adoption and its influences on changes to employees’ behavior, including the extent to which employees require DL. Future research should use theoretical frameworks that predict changes to employees’ abilities, attitudes, motivation, and behavior that result from technology adoption, such as the theory of planned behavior and the technology acceptance model (Choudhary and Bansal, 2022). This would foster the development of strategies that enhance employees’ DL, shape attitudes toward technology, and drive desired behavioral outcomes in digital workplaces (Taherdoost, 2018).

Previously before 2023, most research on DL and employability used qualitative studies, but from 2023 until July 2024, most research used quantitative studies, with only six extant studies of business. To advance understanding of the topic, future research should use mixed methods to assess businesses, especially by comparing employees’ and leaders’/superiors’ responses (Hoedemakers et al., 2023). Such approaches would provide valuable insights into the perspectives and expectations of both employees and managers (Stoffers and Van der Heijden, 2018), allowing more comprehensive analyses of relationships between DL and employability in business. Using mixed methods, researchers can obtain robust data that enable statistical analyses and facilitate a deeper understanding of factors that influence DL and employability in organizations.

DL and IWB research would benefit from qualitative methods (Stoffers et al., 2014), since few such studies examine individual perspectives across stakeholders (Messmann et al., 2017), including society, employees, and organizational leaders. By conducting interviews, focus groups, and case studies, researchers would gain greater insights into factors and contexts that contribute to relationships between DL and IWBs. By understanding individuals’ nuances and experiences in organizational contexts, qualitative research provides valuable recommendations to leaders and organizations that could then leverage DL and thus promote innovation.

Findings from the bibliometric analysis and SLR suggest that relationships among DL, employability, and IWBs in organizations and firms are under-researched. Extant studies suggest influences of employability and IWBs in organizational firms (Stoffers et al., 2020a; Stoffers et al., 2020b; Stoffers et al., 2020c; Stoffers and Van der Heijden, 2018), but none includes DL. Moreover, DL can help employees to deal with rapidly changing environments, especially with the pace of technology development. Through such competencies, employees can deal with technology and use it to achieve innovativeness.

### 4.2 Practical implications

This study contributes to practices, policies, and decision-making of businesses and managers, particularly in the context of a digital workplace. DL has become a crucial factor for employment because it helps future employees to use technology effectively, thereby increasing employability. Labor markets have begun demonstrating that potential workers who are skilled in technology have greater potential to acquire jobs.

Although employees’ degree of DL should be measured properly, varying DL assessment tools, and variations in DL requirements based on job specifications, leave organizations without appropriate measures, and thus DL is often not measured. Organizations should select measurement methods that reflect their unique cultures, job specifications, and other relevant factors. Organizations must support employees’ DL and should provide training and workshops and promote the use of DL so that employees can use it to enhance innovativeness. For example, employees can use computers to search for new information that can help an organization stay informed about industry conditions.

### 4.3 Limitations

Data sources included WoS, Scopus, ProQuest, and EBSCO Academic Search Premier, which means that not all publications on the subject were considered. We urge researchers to conduct more comprehensive investigations by incorporating diverse databases. This study was confined to English-language publications, preventing examination of articles published in other languages. This linguistic limitation suggests the potential exclusion of insights from non-English sources. The study’s scope delineated DL, employability, and IWB. Future research should include alternative terms to further assess the intersection of DL and human resources dynamics, an approach that would deepen understanding of DL’s significance to HRM.

## 5 Conclusion

This study represents a synthesis of research that encompasses HR outcomes of DL, employability, and IWBs, drawing from articles published up to July 2024. By investigating research trends, measurement, conceptual models, and theoretical frameworks, this study unveils essential insights, and we identify promising research directions and gaps that warrant further investigation. Capturing these indicative research paths, our framework serves as a roadmap for future exploration. We thus call for research to intensify focus on contextualized organizational perspectives, employ a diverse spectrum of methodologies, encompass broader variables, use disparate theoretical frameworks, and assess multiple levels of analysis. Such research is both pivotal to the advancement of the field and integral to effective organizational management. To increase the relevance and usefulness of topics, we recommend the use of contexts. This study makes several contributions to broader discourses on DL, employability, and IWBs, demonstrating paths for future scholarly inquiries.

## Data Availability

The original contributions presented in the study are included in the article/supplementary material, further inquiries can be directed to the corresponding author/s.
